# Comprehensive Performance Evaluation of Epoxy Reclaimed Asphalt and Mixtures

**DOI:** 10.3390/ma18050982

**Published:** 2025-02-23

**Authors:** Junhao Tian, Dedong Guo, Qi Xu, Jiang Wu, Xupeng Sun, Li Wang, Chiara Riccardi

**Affiliations:** 1 School of Transportation Civil Engineering, Shandong Jiaotong University, Ji’nan 250357, China; 23107034@stu.sdjtu.edu.cn (J.T.); 22107018@stu.sdjtu.edu.cn (Q.X.); 21107021@stu.sdjtu.edu.cn (J.W.); 22107029@stu.sdjtu.edu.cn (X.S.); 2 Shandong Sanjian Group Co., Ltd., Ji’nan 250100, China; 3 Department of Civil and Industrial Engineering, University of Pisa, Largo Lucio Lazzarino 1, 56126 Pisa, Italy; chiara.riccardi@unipi.it

**Keywords:** RAP, 100% dosage, recycling, microscopic mechanism, epoxy system, road performance

## Abstract

In order to improve the reclaimed asphalt pavement (RAP) dosing and the road performance of recycled asphalt mixtures, this study prepared epoxy recycled binder (ERB) and epoxy recycled mixtures (ERMs) by dosing epoxy asphalt, respectively. The rheological characteristics and microstructure of ERB were comprehensively analyzed using a dynamic shear rheometer (DSR), a bending beam rheometer (BBR), and fluorescence microscopy (FM). The road performance of ERM was evaluated by a four-point bending test, a rutting test, trabecular beam bending test, a freeze–thaw splitting test, an immersion Marshall test, and a uniaxial compression dynamic modulus test. Grey relational analysis (GRA) was used to quantify the correlation between the dosage of epoxy system and road performance indicators. The results show that, after the addition of the epoxy system, the high- and low-temperature rheological properties of ERB were improved by 458.3% and 97.9% compared with those of ordinary asphalt, and the high-temperature performance and fatigue performance of ERM were improved by 220.4% and 80.5% compared with SBS-modified asphalt mixtures. The dynamic modulus test showed that the dynamic modulus of ERM was positively correlated with the dosage of epoxy system. GRA showed that the dosage of epoxy system was most closely related to the fatigue performance of recycled mixtures.

## 1. Introduction

Most of the high-grade highways in China use asphalt pavement; China has now entered into a comprehensive maintenance stage, producing more than 230 million tons of reclaimed asphalt pavement (RAP) annually [1,2,3]. At the same time, highway construction consumes more than 500 million tons of asphalt mixtures every year, and the recycling of RAP is a necessary way to alleviate the scarcity of resources for the construction of transportation infrastructure and protect the natural environment [4,5,6]. As the recycling technology with the widest application range and the highest recycling quality, plant-mixed thermal recycling is widely used all over the world [7,8,9]. However, due to the serious aging of asphalt in RAP, gradation refinement, and heating difficulty, the performance of recycled mixtures gradually declines with the increase in RAP dosage, mainly in the low-temperature cracking resistance, water stability, and poor fatigue performance [10,11,12]. In engineering practice, the mixing rate of plant-mixed hot recycled asphalt mixtures is usually controlled at 20% to 30% [13,14,15], and the low utilization efficiency makes the RAP stock continue to grow. Therefore, in the context of sustainable development strategy, realizing the enhancement of RAP doping and long-term performance guarantee is a difficult problem that needs to be solved at present [16,17,18].

In order to enhance the RAP dosage, domestic and foreign scholars, mainly through the optimization of gradation, change the heating method and mixing modifiers to improve the performance of recycled asphalt mixtures. Wang et al. optimized the gradation by increasing the passing rate of 4.75 mm sieve holes and obtained high-RAP-dosage recycled mixtures with good low-temperature resistance to cracking, but did not explore their long-term durability [19]. Based on a low-RAP-dosage proportion design method and construction process, Zhang applied microwave heating to plant mix thermal regeneration, preparing 50% to 80% RAP dosage regeneration mixtures. In addition to a relatively poor high-temperature performance, the effect of secondary aging of RAP also failed, and the proportion design and construction process was not in line with high-dosage regeneration [20]. Li et al. prepared a high-RAP recycled mixture by mixing SBS-modified asphalt with a recycling agent, but its water stability was not as good as that of ordinary asphalt pavement [21]. It is not difficult to see that the above studies, although they enhanced the mixing of RAP in recycled mixtures to a certain extent, yielded a road performance that was still somewhat inadequate, and RAP enhancement was limited to 100% in ultra-high mixing.

Epoxy asphalt is a high-performance binder made from epoxy resin, a curing agent, and asphalt [22,23,24]. Epoxy resin and the curing agent form a stable three-dimensional network structure through a curing reaction. This can significantly enhance the physicochemical performance of the material, and so the method is widely applied to the paving of steel bridge decks [25,26]. In recent years, in order to somewhat improve the utilization efficiency and regeneration quality of RAP, researchers began to apply epoxy asphalt to the field of RAP regeneration [27,28,29]. In the binder research, Yi et al. simulated aging asphalt in the laboratory and explored the effect of epoxy system doping on the performance of aging asphalt, and the results showed that epoxy resin can significantly improve the performance of aging asphalt [30]. Cheng et al. investigated the influence of the aging degree of asphalt on the performance of epoxy asphalt and found that the aging degree of asphalt has a significant effect on the performance of epoxy asphalt [31]. In the evaluation of mix performance, Chen et al. studied the road performance of 100% RAP dosing epoxy recycled mixtures (ERMs) before and after aging and showed that the mix is more suitable for the construction of the middle and lower layers of the road [32]. Fan et al. investigated the cracking resistance of ERM with 100% RAP dosing and showed that the mixes had better cracking resistance than conventional recycled pavements [33].

It can be seen that the performance of recycled mixtures decreases with the increase in RAP dosage, but epoxy asphalt shows great potential in high-RAP-dosage recycling applications due to its excellent characteristics [34]. The current research focuses on the effect of the epoxy system on the crack resistance of recycled mixtures, but less on its overall pavement performance, and the representativeness of the aging asphalt samples used in the experiments is controversial.

In this study, the aged asphalt extracted from RAP and epoxy asphalt were selected to prepare epoxy recycled binder (ERB), and 100% RAP-doped ERM was prepared by using RAP, 70# matrix asphalt, and an epoxy system. Firstly, the effects of the dosage of epoxy system on the rheological properties and micro-morphology of ERB were investigated. Secondly, the effects of the dosage of epoxy system on the road performance and mechanical performance of ERM were analyzed and compared with those of SBS-modified asphalt mixtures. Finally, grey relational analysis (GRA) quantified the correlation between the dosage of the epoxy system and the road performance indexes. The results of this study are expected to promote the broad application of epoxy asphalt in green road construction and the efficient recycling of RAP.

## 2. Materials and Methods

### 2.1. Materials and Sample Preparation

(1) Epoxy system

**Figure 1:**
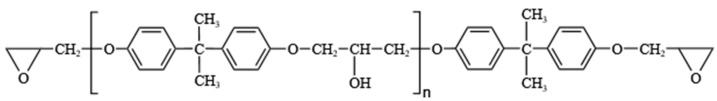
E51 bisphenol A epoxy resin structural formulae.

**Figure 2:**
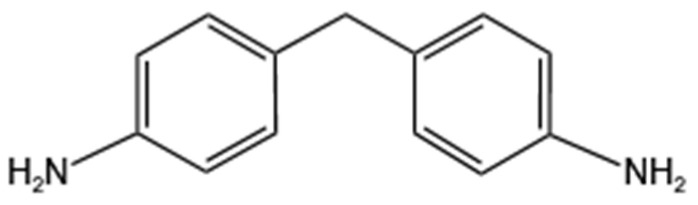
4,4′-diaminodiphenylmethane curing agent structural formulae.

It comprises components A (E51 bisphenol A epoxy resin) (Shanghai Zhanyun Chemical Co., Ltd., Shanghai, China) and B (4,4′-diaminodiphenylmethane curing agent and diluted bitumen) (Wuxi Boruiyu Chemical Technology Co., Ltd., Wuxi, China). The structural formulae of the epoxy resin and curing agent are shown in Figure 1 and Figure 2. Its general technical specifications are shown in Table 1.

(2) Asphalt

SBS-modified asphalt and 70# matrix asphalt were selected for the test, and their basic physical properties are listed in Table 2.

(3) RAP

The test with RAP is divided into coarse and fine grades. Its extraction sieving, respectively, can determine the oil–rock ratio and gradation of RAP. The use of the rotary evaporator method to recover the old asphalt in the RAP and the determination of its three major indicators are shown in Table 3 and Table 4.

(4) New mineral.

Limestone (Shandong Zhanfei Building Materials Co., Ltd., Binzhou, China) was the new mineral used in this experiment, and its main properties are shown in Table 5.

### 2.2. Preparation of Samples

In this experiment, four kinds of epoxy system doping, 10%, 20%, 30%, and 40%, were used to study the properties of ERB and REM. The four ERB samples were ERB10, ERB20, ERB30, and ERB40. The four ERM samples were ERM10, ERM20, ERM30, and ERM40. Their doping schemes are shown in Table 6.

**Figure 3:**
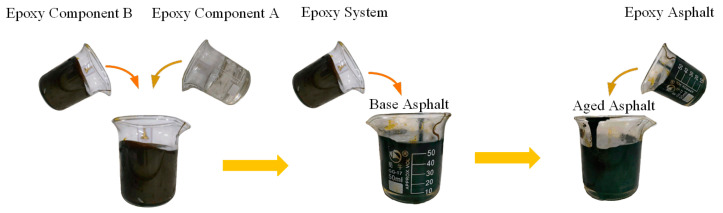
ERB preparation process.

The preparation process of ERB is as follows. (1) Epoxy asphalt was produced by mixing epoxy component A and component B at a mass ratio of 1:3 at 80 °C, followed by mixing with 70# matrix asphalt at 140 °C. (2) The resulting epoxy asphalt was mixed with aged asphalt to produce ERB. (3) The ERB was placed in an oven at 120 °C for complete curing (16 h), cooled to room temperature, and tested. The ERB preparation process is shown in Figure 3.

**Figure 4:**
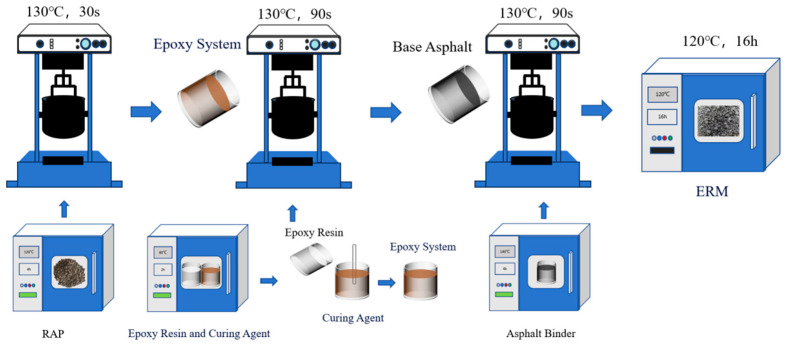
ERM preparation process.

ERM was prepared as follows. (1) RAP was preheated in an oven at 120 °C for more than 4 h, the epoxy system (components A and B) was preheated in an oven at 80 °C for more than 2 h, and 70# matrix asphalt was preheated at 140 °C until it was completely fluid. (2) In the mixing pot at 130 °C, after dry mixing RAP for 30 s, add the preheated epoxy system and mix for 90 s, then add 70# matrix asphalt into the mixing pot and continue mixing for 90 s. (3) To simulate the transportation conditions in actual production, the prepared ERM was held at the discharge temperature for 1 h, and then the specimens were molded. (4) The molded specimens were held in an oven at 120 °C for 16 h until fully cured and cooled to room temperature for subsequent tests. The ERM preparation process is shown in Figure 4.

**Figure 5:**
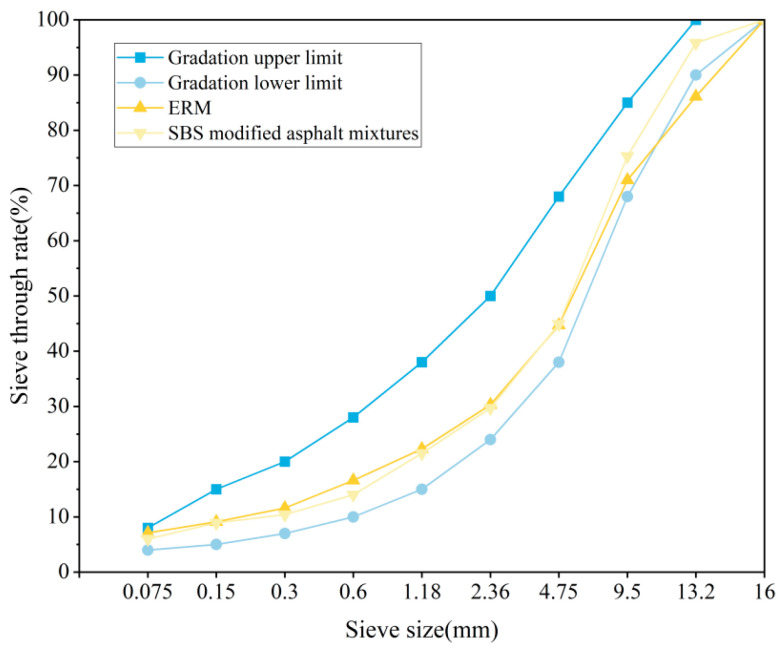
Grading curve.

The 65:35 RAP coarse and fine aggregate blending ratio determined the optimum oil–rock ratio of 2.0% for the new additions, which resulted in a calculated total oil–rock ratio of 4.9% for the ERM. The gradation curves of the ERM- and SBS-modified asphalt mixtures with a 5.6% oil–rock ratio are shown in Figure 5.

### 2.3. Test Methods

(1) Binder test

a. Dynamic shear rheometer (DSR) test

The Gemini II ADS-type DSR from Malvern, UK (Malvern Panalytical Ltd., Worcestershire, UK), set the temperature gradient at 6 °C and the temperature range from 46 °C to 82 °C. The temperature scanning test was performed by selecting the strain control mode with a loading strain level of 10%, a loading frequency of 10 rad/s, a gap of 1 mm, and a parallel plate diameter of 25 mm. The complex modulus G*, phase angle δ and rutting factor G*/sinδ were determined and obtained for four recycled binders. Referring to the relevant standards of the USA “Superpave” specification, such as the rutting factor G*/sinδ ≥ 1 kPa, as the threshold for high-temperature evaluation [36], the advantages and disadvantages of different binders’ high-temperature performance are analyzed by comparing the binders’ rutting factor.

b. Bending beam rheometer (BBR) test

The TE-BBR-F-type low-temperature BBR produced by the United States CANNON Instruments Company was used, and the test temperature was set to −6 °C, −12 °C and −18 °C. As the ERB needs to be cured after molding the specimen, the binder still has a certain mobility at the early stage of curing, and it will trickle out of the test mold. Therefore, a customized high-temperature silicone mold of the same size as the test specimen groove was used as the specimen molding tool. Firstly, the homogeneously mixed recycled binder was poured back and forth at a uniform speed on top of the test mold, with pouring completed when the binder level was slightly higher than the groove. Secondly, while the binder retained fluidity, the mold was immediately scraped and transferred into the oven for curing until the specimen reached room temperature after demolding to obtain the ERB beam specimen. Finally, the specimen was placed into the BBR in a water bath insulation for 1 h, then moved to the bracket, and the indenter load was set to 35 mN. The equipment was operated to test the four different kinds of recycled binder creep strength *S*, creep rate *m*, and obtain the *m*/*S* ratio, referring to the relevant standards of the USA “Superpave” specification, such as *S* ≤ 300 MPa, *m* ≥ 0.3, to determine whether the binder meets the requirements of low-temperature performance.

c. Fluorescence microscopy (FM) test

The FM of Axiocam 506 color type manufactured by Carl Zeiss AG, Germany, was used to observe the microphase structure of ERB with different dosages of the epoxy system (10%, 20%, 30%, and 40%). The procedure was as follows. (1) The slides and coverslips were cleaned with alcohol. (2) The prepared thermally flowable ERB was uniformly stirred and dropped onto the center of the slide, and the coverslip was quickly covered, cured, and cooled to room temperature. (3) The samples were observed under the FM at 100 times magnification, and images were obtained.

The fluorescence micrographs were converted to 8-bit grayscale using “ImageJ” software (version 1.53t, National Institutes of Health, Bethesda, MD, USA), the contrast was adjusted, and the “Default” algorithm was used to perform threshold segmentation, fill the holes, and segment the adherent particles. The volume fraction of the dispersed phase was calculated.

d. Differential scanning calorimeter (DSC) test

**Figure 6:**
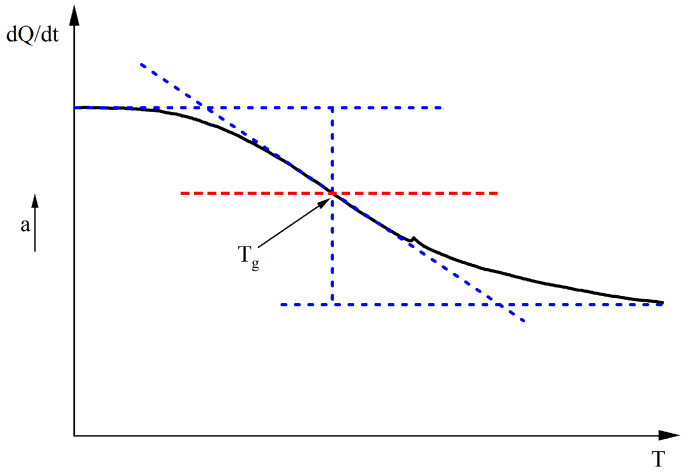
Glass transition temperature calculation method.

The glass transition temperature (T_g_) of 70# matrix asphalt, ERB10, ERB20, ERB30, and ERB40 was measured using a differential scanning calorimeter (DSC-3) manufactured by METTLER TOLEDO, USA. Firstly, the scanning temperature interval of DSC was set to −40 °C to 0 °C, and the heating rate was set to 10 °C/min. Secondly, the DSC curves were obtained by scanning the five binder materials. Finally, as shown in Figure 6, a straight line parallel to the two baselines before and after the transition was made, and the temperature corresponding to the intersection of the line and the curve was the glass transition temperature.

(2) Mixture test

a. Conventional road performance test

By the relevant provisions of the “Test Procedures for Asphalt and Bituminous Mixtures in Highway Engineering”(JTG E20-2011), four-point bending test, rutting test, trabecular beam bending test, freeze–thaw splitting test, immersion Marshall test, and uniaxial compression dynamic modulus test were carried out on SBS-modified asphalt mixtures, ERM10, ERM20, ERM30, and ERM40, in order to evaluate their conventional road performance.

b. Uniaxial compression dynamic modulus test

Referring to the American AASHTO T378-17(TP79) standard and the relevant requirements of “Test Procedures for Asphalt and Bituminous Mixtures in Highway Engineering” (JTG E20-2011) T0378-2011, the mechanical properties of the mixtures were evaluated by uniaxial compression dynamic modulus test. The size of the test specimen was a cylinder with a height of 150 mm and a diameter of 100 mm, and the set temperatures were 5 °C, 20 °C, 35 °C, and 50 °C. The temperature was set at 5 °C, 20 °C, 35 °C, and 50 °C, and the continuous half-sine waveform was selected, and the loading frequency was 0.1 Hz, 0.5 Hz, 1 Hz, 5 Hz, 10 Hz, and 25 Hz.

Based on the principle of time-temperature equivalence, 35 °C was selected as the reference temperature. The dynamic modulus curves at other temperatures, shifted left and right along the horizontal direction by a certain distance, were overlapped with the modulus curves at the reference temperature to obtain the dynamic modulus master curves, which were then fitted using the Sigmoidal mathematical model Equation (1).

(1) $$lg|{Ε}^{*}|=\delta +\frac{\alpha }{1+e^{\beta +\gamma •\mathrm{lg}t_{r}}}$$

(3) GRA

GRA is a mathematical method to explore the interrelationships between factors within a system [37]. The method primarily assesses and compares the degree of similarity or association between different data series. The following outlines the basic steps of GRA:

a. Identification of reference and comparison series.

(2) $$X_{0}=\left\{X_{0}\left(k\right)|k=1,2,\cdots ,n\right\}$$

b. The variables are dimensionless; here, the initial value method is applied.

(3) $$X_{i}=\left\{X_{i}\left(k\right)\left|k=1,2,\cdots ,n\right.\right\},\left(i=1,2,\cdots ,n\right)$$

c. Find the sequence of differences, the maximum difference and the minimum difference.

(4) $$\left\{\begin{array}{l} Y_{0}=\left\{X_{0}(k)/{\bar{X}}_{0}|k=1,2,\cdots ,n\right\}\\ Y_{i}=\left\{X_{i}\left(k\right)/\bar{X_{i}}|k=1,2,\cdots ,n\right\},\left(i=1,2,\cdots ,n\right) \end{array}\right.$$

d. Calculation of correlation coefficients.

(5) $$\xi_{i}=\frac{\underset{i=1,n}{min}\underset{k=1,n}{min}\left|Y_{0}\left(k\right)-Y_{i}\left(k\right)\right|+\rho \underset{i=1,n}{max}\underset{k=1,n}{max}\left|Y_{0}(k)-Y_{i}(k)\right|}{\left|Y_{0}(k)-Y_{i}(k)\right|+\rho \underset{i=1,n}{max}\underset{k=1,n}{max}\left|Y_{0}(k)-Y_{i}(k)\right|}$$

e. Finding correlations.

(6) $$\gamma_{i}=\frac{1}{n}\sum_{k=1}^{n}\xi_{i}(k)$$

## 3. Results

### 3.1. ERB Properties

#### 3.1.1. Rheological Properties

(1) High-temperature performance

**Figure 7:**
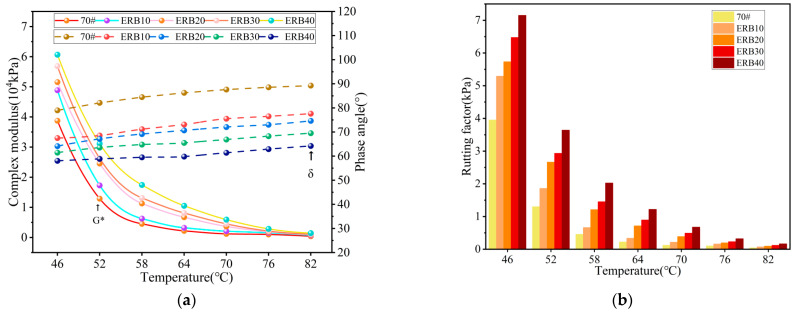
ERB high-temperature performance test results. (**a**) Complex shear modulus and phase angle; (**b**) rutting factor.

The complex shear modulus, phase angle, and rutting factor of ERB10, ERB20, ERB30, and ERB40 at 46 °C to 82 °C were determined, and the test results are shown in Figure 7.

The introduction of the epoxy system improved the complex modulus of ERB, which showed that at the same temperature, the complex modulus of ERB with different dosages of the epoxy system exceeded that of ordinary asphalt, and the modulus increase was more significant with the increase in dosage. It can be attributed to the fact that the epoxy system introduces thermosetting components into the binder through the curing reaction, which enhances the structural strength and reduces the sensitivity to high temperatures. For example, at 82 °C, the epoxy system in the range of 10% to 40% can increase the complex modulus of recycled binders by 71.4% to 254.8% over that of new asphalt. At the same temperature, recycled binders with higher epoxy system dosage have a lower phase angle, indicating that they maintain better elasticity at high temperatures, reduce plastic deformation, and improve high-temperature performance. For example, at 82 °C, the phase angle can be reduced by 13.1% to 28.1% when the dosage of the epoxy system is increased compared with that of ordinary asphalt.

Analysis of the rutting factor shows that, at the same temperature, for different dosages of epoxy system, the recycled asphalt rutting factor is significantly higher than that of ordinary asphalt, which reflects that the epoxy system has high-strength and high-elasticity characteristics, and can effectively reduce the temperature sensitivity of the recycled asphalt, on the whole, to improve the high-temperature performance of recycled asphalt. In the temperature range of 46 °C to 82 °C, when the epoxy system doping is between 10% and 40%, its rutting factor compared to ordinary asphalt can be improved up to 458.3%.

(2) Low-temperature performance

**Figure 8:**
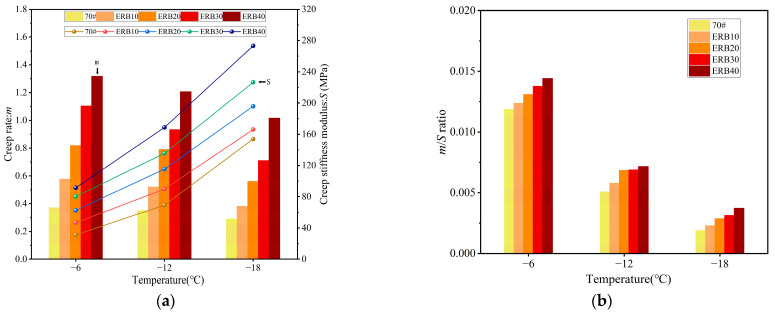
ERB low-temperature performance test results. (**a**) Creep stiffness modulus *S* and creep rate *m*; (**b**) *m*/*S* ratio.

The creep stiffness modulus *S*, creep rate *m*, and *m*/*S* ratio of ERB10, ERB20, ERB30, and ERB40 were determined at −6 °C, −12 °C, and −18 °C, and the test results are shown in Figure 8.

The increase in the dosage of the epoxy system led to an increase in the creep strength of ERB, indicating that the increase in the dosage reduced the deformation capacity of ERB at low temperatures. The creep strength of ERB was improved by 7.8% to 77.4% compared with that of ordinary asphalt at the same temperature of −18 °C with the increase in the dosage of the epoxy system (10% to 40%). At the same time, the increase in the dosage of the epoxy system also promoted the increase in the creep rate of ERB, which in turn improved the stress relaxation ability of the material. At −18 °C, the ERB creep rates of different epoxy system dosages were enhanced by 31% to 250.3% compared with 70# matrix asphalt.

Generally, a smaller *S* and a larger *m* indicate better low-temperature binder performance under identical low-temperature conditions. However, evaluating low-temperature performance solely based on either *S* or *m* is unreasonable. A comprehensive analysis should be conducted by simultaneously considering both parameters when assessing recycled binders’ low-temperature performance. Therefore, this study introduces the *m*/*S* ratio as an evaluation metric, where a higher *m*/*S* ratio corresponds to superior low-temperature performance. The analysis of Figure 8b shows that at the same temperature, as the doping of the epoxy system increases, the larger the ratio of *m*/*S*, the better the low-temperature performance of ERB. It is because although the increase in epoxy system doping reduces the low-temperature deformation capacity of the recycled binder, it also significantly improves the stress relaxation capacity of the binder, and the degree of enhancement is much higher than the degree of reduction in low-temperature deformation capacity. In the temperature range of −18 °C to −6 °C, the *m/S* was improved by up to 97.9% compared to the new asphalt when the epoxy system was dosed between 10% and 40%.

#### 3.1.2. Microscopic Analysis

**Figure 9:**
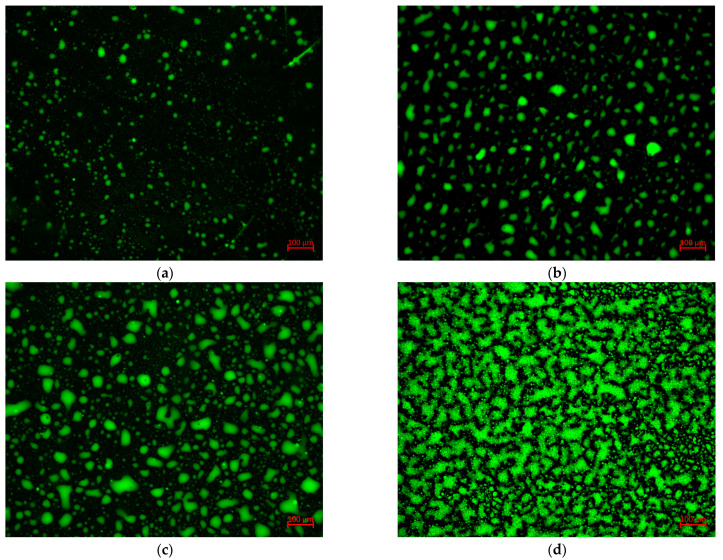
Fluorescence microscope test results. (**a**) ERB10; (**b**) ERB20; (**c**) ERB30; (**d**) ERB40.

**Figure 10:**
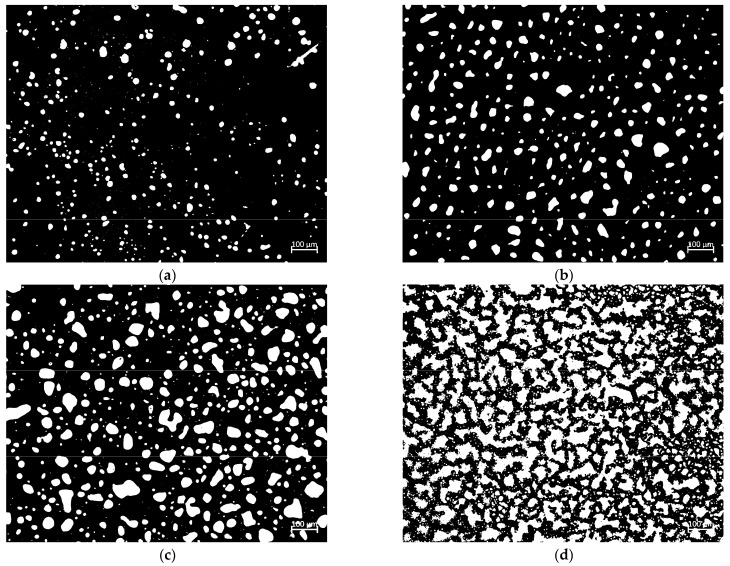
FM image processed by ImageJ threshold segmentation. (**a**) ERB10; (**b**) ERB20; (**c**) ERB30; (**d**) ERB40.

The microphase structure of the ERB with different dosages of the epoxy system (10%, 20%, 30%, and 40%) was observed using an Axiocam 506 color fluorescence microscope, and the results are shown in Figure 9. The FM images processed by “ImageJ” threshold segmentation are shown in Figure 10.

The volume fractions of the dispersed phase of ERB10, ERB20, ERB30, and ERB40 were calculated to be 6.73%, 17.63%, 29.76%, and 41.23%, respectively. Under 10% epoxy system doping, the volume fraction of ERB is smaller than the initial concentration of epoxy resin, indicating that part of the epoxy resin is dissolved in the asphalt matrix. At this time, the asphalt is a continuous phase, the epoxy system is a dispersed phase, and the epoxy system is dispersed in the ERB as a single particle, which does not form a complete cross-linking structure. When the dosage of the epoxy system is 20%, although part of the epoxy resin is still dissolved in the asphalt matrix, the fluorescence microscopic image clearly shows that the epoxy system in the form of particles began to produce local cross-linking, and the dispersed phase of the epoxy system gradually increases. When the doping of the epoxy system reaches 30%, the volume fraction of the dispersed phase of the epoxy system is close to the proportion of the continuous phase of the asphalt, and the epoxy system produces obvious cross-linking, indicating that the system is gradually transitioning to the bicontinuous phase structure. When the dosage of the epoxy system reaches 40%, the volume fraction of the dispersed phase of ERB exceeds the initial concentration of epoxy resin. Combined with the DSC later in the article, it can be inferred that cross-linking-induced phase separation (CIPS) occurs during the curing process, with the epoxy resin cross-linking to form a continuous phase and the asphalt being split into a dispersed phase.

In conclusion, the addition of the epoxy system can make the formation of a stable three-dimensional network structure between epoxy resin and asphalt, improve the rheological properties of ERB, and enhance the performance of the mix.

#### 3.1.3. Glass Transition Temperature

**Figure 11:**
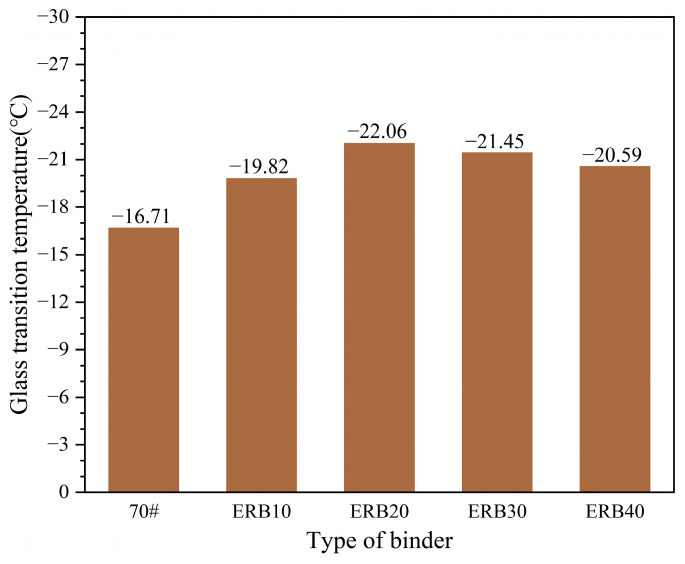
Glass transition temperature test results.

The glass transition temperature on the DSC curves of the five binders (70# matrix asphalt, ERB10, ERB20, ERB30, and ERB40) was calculated, and the data were collected, as shown in Figure 11.

It is well known that the glass transition temperature of asphalt increases significantly after aging, but observation of Figure 11 shows that the glass transition temperature of the four ERBs after the introduction of epoxy resin is lower than that of the 70# matrix asphalt, which proves that the epoxy resin can indeed improve the mechanical properties of asphalt. The reason for this is analyzed because of the affinity between epoxy resin and polar asphaltene groups in the asphalt. The epoxy resin is adsorbed in multiple layers on the surface of the asphalt, and this adsorption improves the mobility of nonpolar molecular chains in the soft asphaltene and decreases the glass transition temperature.

With the increase in epoxy doping, the glass transition temperature of ERB should be lower and lower, but after the glass transition temperature of ERB20 decreased to −22.06 °C, the glass transition temperature of ERB30 and ERB40 increased. The reason for this is that after the increase in the dosage of the epoxy system, although more epoxy resin is adsorbed on the surface of the asphalt in the multilayer, the three-dimensional network structure formed by the curing of the epoxy resin also increases. The three-dimensional network structure wraps the asphalt molecules, restricting the movement of some of the nonpolar molecular chains, which ultimately increases the glass transition temperature.

### 3.2. ERM Properties

**Figure 12:**
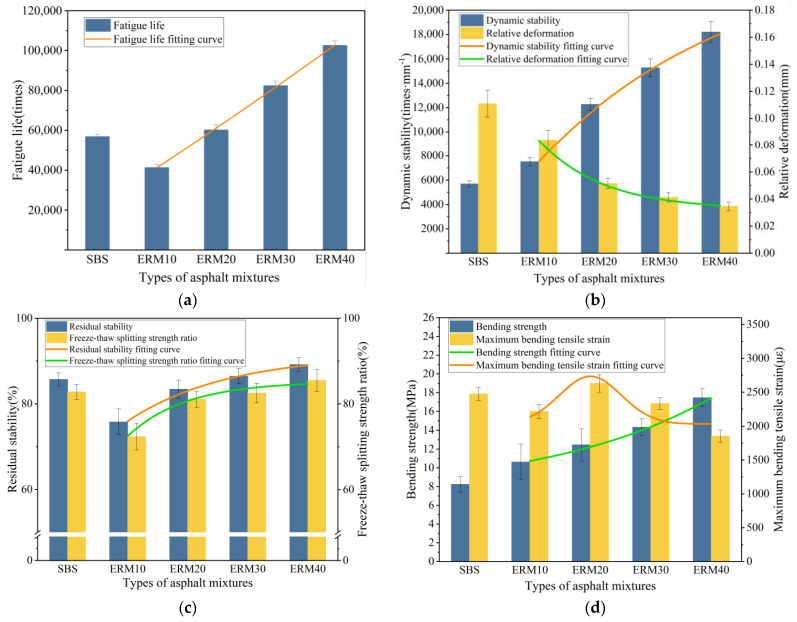
Conventional road performance test results. (**a**) Fatigue performance; (**b**) high-temperature performance; (**c**) water stability; (**d**) low-temperature cracking resistance.

The comparison results of the conventional performance of ERM and SBS-modified asphalt mixtures with four different epoxy systems (ERM10, ERM20, ERM30, and ERM40) are shown in Figure 12.

Fatigue life is an important index to evaluate the long-term service life of asphalt mixtures. From Figure 12a, it is obvious that the fatigue life enhancement of ERM at 20% dosing is the most significant at 31.35%, but ERM30 and ERM40 have better fatigue life. Dynamic stability is an important index to evaluate the rutting resistance of asphalt mixtures. From Figure 12b, it can be seen that the dynamic stability of ERM increases with the increase in the dosage of the epoxy system, and the most significant increase is 38.75% at 20% dosage. The relative deformation of ERM decreases, which is better than that of SBS-modified asphalt mixtures. This means that ERM has stronger deformation resistance at high temperatures and is less prone to rutting. It not only extends the pavement’s service life but also reduces the maintenance cost of the pavement. Residual stability is an important index for evaluating the ability of asphalt mixtures to maintain performance under water damage conditions. From Figure 12c, it can be seen that although the residual stability and freeze–thaw splitting strength ratio of ERM with 20% and lower epoxy system dosage is not as good as that of SBS-modified asphalt mixtures, ERM with 30% and higher epoxy system dosage has stronger water damage resistance and durability than SBS-modified asphalt mixtures. This not only significantly reduces pavement maintenance and repair costs but also greatly improves pavement comfort. Flexural strength and maximum flexural strain are important indicators for evaluating the low-temperature performance of asphalt mixtures. As can be seen from Figure 12d, the bending strength of ERM is positively correlated with the dosage of the epoxy system. However, the maximum bending tensile strain reaches the highest at 20% dosage of the epoxy system and decreases from 30% dosage with a general tendency of increasing first and then decreasing.

In summary, the performance of ERM20 is more potent than that of SBS-modified asphalt mixtures. However, for ERM30, except for the low-temperature crack resistance, which is not as good as SBS-modified asphalt mixtures, the performance is much better than ERM20. The best epoxy system dosage is 20% for the comprehensive construction economy and practicality.

### 3.3. Mechanical Response

#### 3.3.1. Dynamic Modulus

**Figure 13:**
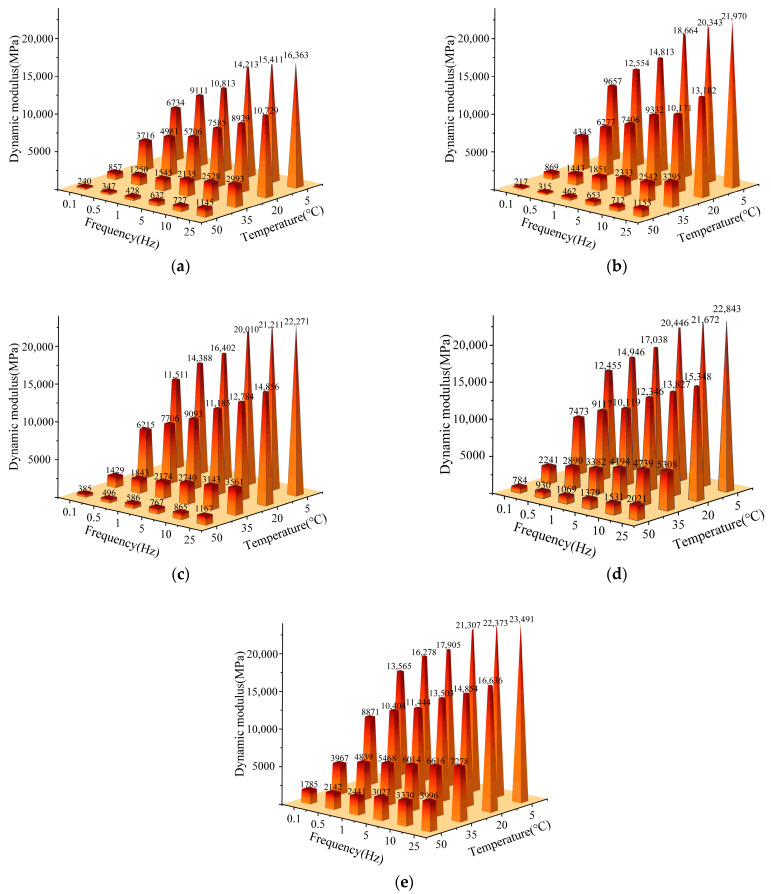
Dynamic modulus test results. (**a**) SBS-modified asphalt mixtures; (**b**) ERM10; (**c**) ERM20; (**d**) ERM30; (**e**) ERM40.

Four ERM- and SBS-modified asphalt mixtures were subjected to dynamic modulus tests, and SBS-modified asphalt mixtures were analyzed compared to ERM. The results are shown in Figure 13.

Under the experimental conditions of 20 °C and 10 Hz, the dynamic modulus of ERM increased by 13.9%, 43.2%, 54.9%, and 66.4% with the increase in the dosage of the epoxy system to reach a maximum value of 14,854 MPa, which is 1.66 times the modulus observed in SBS-modified asphalt mixtures. These results indicate that ERM has superior deformation resistance. However, the potential effects of modulus enhancement on pavement performance should be considered in the design of pavement structures.

Further observation of the dynamic modulus change of ERM- and SBS-modified asphalt mixtures at different frequencies (10 Hz and 5 Hz) shows that ERM’s modulus change decreases when the epoxy system’s dosage is more than 20%. This indicates that the epoxy recycled asphalt pavement can provide more stable performance under the conditions of speed variation. In addition, by comparing the dynamic modulus at different temperatures, ERM exhibits lower temperature sensitivity and more minor modulus changes under different temperature conditions, showing the superiority of ERM in terms of temperature stability.

#### 3.3.2. Dynamic Modulus Master Curve

**Figure 14:**
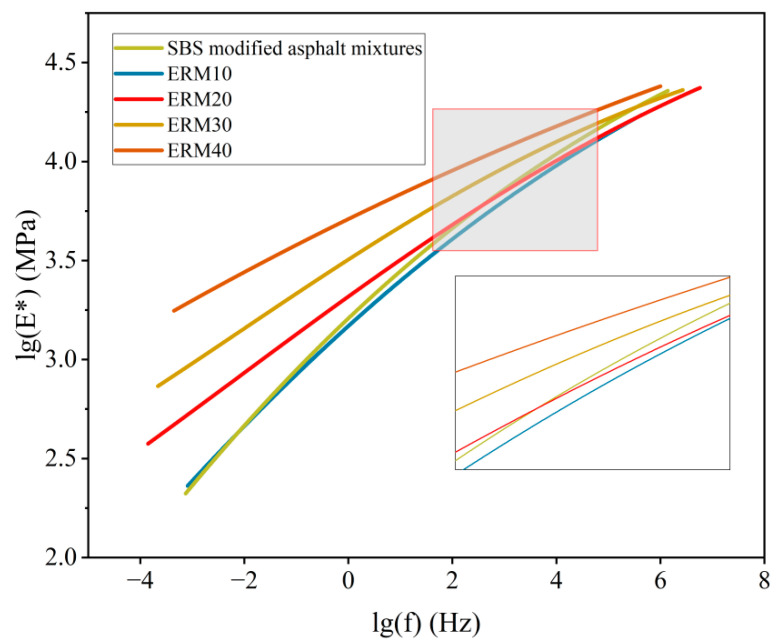
Dynamic modulus master curve.

According to the dynamic modulus test results, the dynamic modulus master curves of SBS-modified asphalt mixtures and ERM10, ERM20, ERM30, and ERM40 were made, as shown in Figure 14.

In this study, ERM doped with a high amount of epoxy system exhibited generally higher master curves than ERM doped with a low amount of epoxy system. The reasons for the analysis can be attributed to two aspects. Firstly, the light component of the aged asphalt in the RAP is reduced, while the heavy component is increased, which enhances the stability of the colloidal structure; secondly, the strength of epoxy asphalt, as a thermosetting material, is enhanced with the increase in doping, and the increase in crosslink density further strengthens the mechanical properties of ERM.

For the ERM doped with four different epoxy systems, the slopes of the main curves of dynamic modulus decreased with increasing frequency. This can be attributed to the fact that the loading time is longer under low-frequency conditions, and the asphalt mixtures have more time to respond to the loading, resulting in larger deformation and relatively lower modulus. Under high-frequency conditions, the loading time is short, and the material’s response tends to be more toward transient elastic behavior, thus exhibiting a higher modulus and lower slope.

### 3.4. GRA

**Figure 15:**
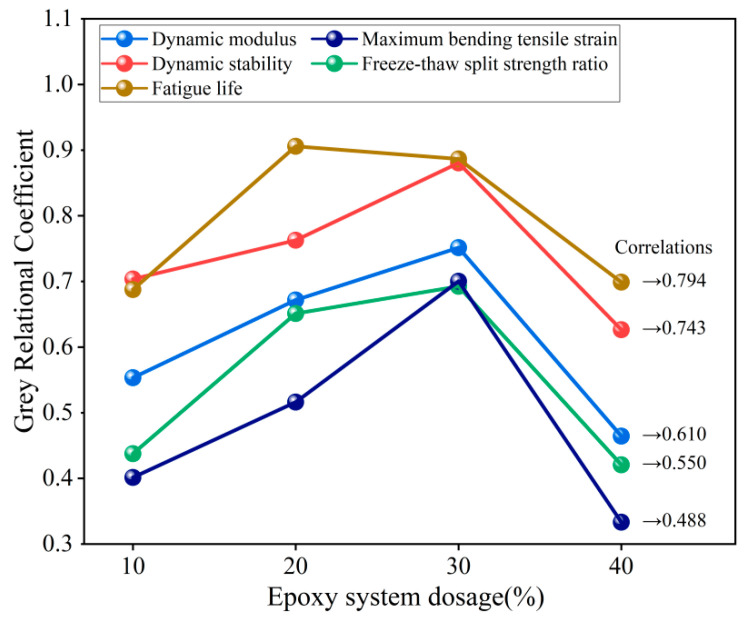
GRA test results.

The dosage of the epoxy system was used as the reference series, and the performance evaluation indexes, such as dynamic modulus, fatigue life, dynamic stability, maximum bending and tensile strain, and freeze–thaw splitting tensile strength ratio, were selected as the comparative series. The GRA was performed on the ERM, as shown in Figure 15.

Epoxy system dosage was most strongly associated with ERM fatigue life, followed by dynamic stability, dynamic modulus, freeze–thaw splitting tensile strength ratio, and the lowest degree of association was with maximum bending and tensile strains. The reason for this analysis is the inherent physico-mechanical advantages of epoxy asphalt, including excellent strength, durability, and adhesion, which enhances the durability of ERM. Because epoxy asphalt is a thermosetting material, the increase in dosage directly affects the high-temperature stability of ERM [38].

## 4. Conclusions

In this study, 100% recycling of RAP by epoxy asphalt was carried out, and the effects of epoxy system dosing (10%, 20%, 30%, 40%) on its performance were investigated from binder to mix. The correlation between the dosing of the epoxy system and road performance indicators was quantified in conjunction with GRA.

(1) Through the DSR test and BBR test, it can be seen that ERB’s high-temperature and low-temperature rheological properties are significantly better than those of ordinary asphalt. In the range of 46 °C to 82 °C, ERB rutting factor, compared to ordinary asphalt, can be improved by up to 458.3%; in the range of −18 °C to −6 °C, ERB creep rate and creep strength of the ratio of ordinary asphalt can be improved by up to 97.9%.

(2) The FM test shows that when the doping of the epoxy system reaches 20% and above, the epoxy system and asphalt directly form a stable three-dimensional network structure. With the increase in doping, the epoxy system becomes more dense, which effectively improves the rheological properties of ERB as well as the road performance of ERM.

(3) With the increase in the dosage of the epoxy system, the high-temperature performance and fatigue performance of ERM were improved by 220.4% and 80.5% compared with SBS-modified asphalt mixtures, and the water stability was also slightly improved.

(4) Regarding low-temperature cracking resistance, ERM’s maximum bending and tensile strains showed an increasing and decreasing trend, reaching the highest at 20% epoxy system dosage. However, for ERM with 30% epoxy system dosage, except for the low-temperature cracking resistance, which is not as good as ERM20, the other properties are excellent, so considering the construction cost and the performance of the mixture, the best epoxy system dosage is 20%.

(5) The dynamic modulus of the four ERMs and their main curves was higher than that of SBS-modified asphalt mixtures, and the dynamic modulus of the ERM was positively correlated with the dosage of the epoxy system. Under 20 °C and 10 Hz conditions, the dynamic modulus can reach up to 14,854 MPa, 1.66 times higher than SBS-modified asphalt mixtures. The possible effects of this modulus difference should be fully considered in the structural design of pavements.

(6) The GRA results showed the highest correlation between the epoxy system dosage and ERM’s fatigue life, which was 0.794, followed by the dynamic stability index of 0.743. The lowest correlation of the maximum bending and tensile strains was 0.488. The fatigue and high-temperature performance of ERM were the most sensitive to the changes in the dosage of the epoxy system.

This study provides a preliminary analysis of epoxy recycled asphalt and its mixes from both macro and micro perspectives. Future research will focus on two aspects: (1) further clarifying the effects of water and freeze–thaw cycles on epoxy recycled asphalt and its mixes; (2) conducting long-term road performance tests on ERM to evaluate their long-term durability.

## Figures and Tables

**Table 1:** General technical specifications of epoxy A and B components.

Components	Experiment Index	Unit	Test Results
Component A	Viscosity(25 °C)	Pa·s	1.8
	Epoxy equivalent	/	209
	Specific gravity(25 °C)	/	1.15
	Appearance	/	Colorless transparent liquid
Component B	Viscosity(25 °C)	Pa·s	5.2
	Specific gravity(25 °C)	/	0.89
	Appearance	/	Amber liquid

**Table 2:** Basic physical properties of SBS-modified asphalt and 70# matrix asphalt.

Physical Performance Indicators	Test Results		Test Methods
	SBS-Modified Asphalt	70# Matrix Asphalt	
Penetration (25 °C, 0.1 mm)	52	71	JTG E20-2011 T 0604 [35]
Softening point (°C)	86.2	47.0	JTG E20-2011 T 0606 [35]
Ductility (15 °C, cm)	31	>100	JTG E20-2011 T 0605 [35]
Viscosity (135 °C, Pa·s)	3.250	0.441	JTG E20-2011 T 0625 [35]

**Table 3:** RAP sieving results and oil–rock ratio.

RAP	The Pass Rate of the Following Sieve Holes (%)										Oil–Rock Ratio (%)
	16	13.2	9.5	4.75	2.36	1.18	0.6	0.3	0.15	0.075	
Coarse aggregate	95.0	78.6	55.4	24.9	17.0	13.2	10.6	7.9	6.2	4.8	2.00
Fine aggregate	100	100	100	81.4	55.1	39.1	27.7	18.5	14.4	11.5	4.58

**Table 4:** Three major indicators of old asphalt.

Parameter Indicators	Penetration (0.1 mm)	Softening Point (°C)	Ductility (10 °C, cm)
Test value	40.3	50.3	9.1

**Table 5:** Main properties of new mineral.

Items	Unit	Data			
		Filler	0–5 mm	5–10 mm	10–15 mm
Crushing value	%	/	/	/	14.5
Needle and flake content	%	/	/	17.2	12.2
Apparent relative density	/	2.721	2.773	2.890	2.751
Gross volume relative density	/	/	/	2.750	2.712
Water absorption	%	/	/	1.21	0.41

**Table 6:** Epoxy system mixing program.

Oil–Rock Ratio (%)	Type of Recycled Mixtures	Newly Added Binder Group Ratio	Mixing Ratio of Old and New Binder After Mixing	
		Epoxy System: Asphalt	Epoxy System: New Asphalt: Old Asphalt	Epoxy System: (New Asphalt + Old Asphalt)
2.00	ERM40	98:2	40:1:59	40:60
	ERM30	73:27	30:11:59	30:70
	ERM20	49:51	20:31:59	20:80
	ERM10	24:76	10:31:59	10:90

## Data Availability

The original contributions presented in this study are included in the article. Further inquiries can be directed to the corresponding author.

## References

[1] Wang Z., Lu W., Liu K., Lv S., Peng X., Yang S., Ding S. (2023). Research on failure strength master curve and fatigue performance of asphalt mixture containing high-proportion reclaimed asphalt pavement. Constr. Build. Mater..

[2] Wang T., Jiang W., Xiao J., Guo D., Yuan D., Wu W., Wang W. (2022). Study on the blending behavior of asphalt binder in mixing process of hot recycling. Case Stud. Constr. Mater..

[3] Li D., Leng Z., Zhang S., Jiang J., Yu H., Wellner F., Leischner S. (2022). Blending efficiency of reclaimed asphalt rubber pavement mixture and its correlation with cracking resistance. RCR Adv..

[4] Liu Q., Han B., Wang S., Falchetto A.C., Wang D., Yu B., Zhang J. (2022). Evaluation and molecular interaction of asphalt modified by rubber particles and used engine oil. J. Clean. Prod..

[5] Xiao F., Xu L., Zhao Z., Hou X. (2023). Recent applications and developments of reclaimed asphalt pavement in China, 2010–2021. Sustain. Mater. Technol..

[6] Wang T., Riccardi C., Wei J. (2025). From waste to sustainable pavement: Rejuvenation of asphalt binder using waste engine oil residue and crumb rubber. Chem. Eng. J..

[7] Renken P., Büchler S., Falchetto A.C., Wang D., Wistuba M.P. (2018). Warm Mix Asphalt-A German Case study. Asph. Paving Technol..

[8] Sha A., Jiang W., Shan J., Wu W., Li Y., Zhang S. (2022). Pavement structure and materials design for sea-crossing bridges and tunnel: Case study of the Hong Kong–Zhuhai–Macau Bridge. J. Road Eng..

[9] Büchner J., Wistuba M.P., Remmler T., Wang D. (2019). On low temperature binder testing using DSR 4 mm geometry. Mater. Struct..

[10] Zhang F., Falchetto A.C., Yuan D., Wang W., Wang D., Sun Y. (2024). Research on performance variations of different asphalt binders results from microwave heating during freeze-thaw cycles. Constr. Build. Mater..

[11] Walther A., Büchler S., Falchetto A.C., Wang D., Riccardi C., Wistuba M.P. (2019). Experimental investigation on asphalt mixtures prepared with reclaimed asphalt pavement and rejuvenators based on the BTSV method. Road Mater. Pavement Des..

[12] Li M., Xing C., Liu L., Huang W., Meng Y. (2022). Gel permeation chromatography-based method for assessing the properties of binders in reclaimed asphalt pavement mixtures. Constr. Build. Mater..

[13] Jameel M.S., Khan A.H., Rehman Z.U., Tarar M.A. (2023). Evaluation of performance characteristics of asphalt mixtures modified with renewable oils and reclaimed asphalt pavement (RAP). Constr. Build. Mater..

[14] Ma X., Wang J., Xu Y. (2022). Investigation on the Effects of RAP Proportions on the Pavement Performance of Recycled Asphalt Mixtures. Front. Mater..

[15] Lamba N., Raj R., Singh P. (2022). Mechanical characteristics of high strength concrete incorporating recycled CFRP fibers. J. Appl. Polym. Sci..

[16] Jiang W., Wang T., Yuan D., Sha A., Zhang S., Zhang Y., Xiao J., Xing C. (2024). Available solar resources and photovoltaic system planning strategy for highway. Renew. Sustain. Energy Rev..

[17] Zhang F., Wang D., Falchetto A.C., Cao Y. (2024). Microwave deicing properties and carbon emissions assessment of asphalt mixtures containing steel slag towards resource conservation and waste reuse. Sci. Total Environ..

[18] Wu W., Cavalli M.C., Jiang W., Kringos N. (2024). Differing perspectives on the use of high-content SBS polymer-modified bitumen. Constr. Build. Mater..

[19] Wang S., Wu C., Zhao Y., Su Z., Su G., Tang D., Yang T. (2024). Analysis of Factors Influencing the Low-Temperature Behavior of Recycled Asphalt Mixtures in Seasonal Freeze-Thaw Regions. Buildings.

[20] Zhang Q.X. (2022). Study on the Road Performance of Microwave Factory Mixed High Content RAP Hot Recycled Asphalt Mixture. Ph.D. Thesis.

[21] Li Y., Li J., Yang H., Ye Z., Hu C., Shen S. (2024). Laboratory evaluation of synergistic blending with SBS-modified bitumen and rejuvenator to enhance the performance of recycled bitumen with a high content of RAP materials. Constr. Build. Mater..

[22] Xie H., Li C., Wang Q. (2022). A critical review on performance and phase separation of thermosetting epoxy asphalt binders and bond coats. Constr. Build. Mater..

[23] Wang Q., Min Z., Wong Y.D., Li M., Huang W. (2023). Evaluation of properties and aging resistance of epoxy asphalt composite modified by ultraviolet absorber. J. Appl. Polym. Sci..

[24] Zhang L., Cheng L., Lu Q., Zhang Z. (2024). Quantitative evaluation of asphalt blending characteristics in epoxy-modified hot recycled asphalt mixtures based on 3D confocal fluorescence technology. J. Appl. Polym. Sci..

[25] Nie W., Wang D., Sun Y., Xu W., Xiao X. (2021). Integrated Design of Structure and Material of Epoxy Asphalt Mixture Used in Steel Bridge Deck Pavement. Buildings.

[26] Wu W., Jiang W., Xiao J., Yuan D., Wang T., Ling X. (2024). Investigation of LAS-based fatigue evaluation methods for high-viscosity modified asphalt binders with high-content polymers. Constr. Build. Mater..

[27] Gao J., Yang J., Yu D., Jiang Y., Ruan K., Tao W., Sun C., Luo L. (2021). Reducing the variability of multi-source reclaimed asphalt pavement materials: A practice in China. Constr. Build. Mater..

[28] Imaninasab R., Loria-Salazar L., Carter A. (2022). Integrated performance evaluation of asphalt mixtures with very high reclaimed asphalt pavement (RAP) content. Constr. Build. Mater..

[29] Chen X., Chen Y., Ma T., Gu L., Shi S. (2024). Study on the performances of epoxy asphalt binders influenced by the dosage of epoxy resin and its application to steel bridge deck pavement. Constr. Build. Mater..

[30] Yi X., Wong Y.D., Chen H., Fan Y., Yang J., Huang W., Wang H. (2023). Influence of epoxy resin polymer on recycled asphalt binder properties. Constr. Build. Mater..

[31] Cheng L., Zhang L., Zhang F., Zhang D., Ma Y. (2023). Evaluation of the effects of asphalt binder aging degree on the curing, compatibility, and mechanical behaviors of epoxy asphalt binders. Constr. Build. Mater..

[32] Chen Y., Wang Y., Guo S., Zhao J., Feng D., Yi J. (2023). Research on aging performance of hot mix fully recycled epoxy asphalt mixture with 100% RAP utilization-Take the surface course as the application target. Constr. Build. Mater..

[33] Fan Y., Chen H., Yi X., Xu G., Cai X., Zhou Y., Huang S., Wu Y., Wang H., Yang J. (2023). Cracking resistance evaluation of epoxy asphalt mixtures with 100% reclaimed asphalt pavement (RAP). Constr. Build. Mater..

[34] Guo D., Sun X., Tian J., Xu M., Yang S., Zou H., Li J., Wang T., Chiara R. (2025). Salt release and performance of self-ice-melting epoxy asphalt pavement under accelerated loading simulation conditions. Constr. Build. Mater..

[35] (1984). Standard Test Methods of Bitumen and Bituminous Mixtures for Highway Engineering.

[36] Sassan A., Pouria H. (2012). Implementing viscoelastic rheological methods to evaluate low temperature performance of modified asphalt binders. Constr. Build. Mater..

[37] Lu P., Huang S., Shen Y., Wu Y., Li D. (2023). Mix design of asphalt plug joint based on response surface method and grey relational analysis. Int. J. Pavement Eng..

[38] Kim T.W., Baek J., Lee H.J., Choi J.Y. (2013). Fatigue performance evaluation of SBS modified mastic asphalt mixtures. Constr. Build. Mater..

